# Supplementary material for: Relationship between trust and patient involvement in medical decision-making: A cross-sectional study
Source: PLoS One. 2021 Aug 26;16(8):e0256698. doi: 10.1371/journal.pone.0256698 (PMC8389380; doi:10.1371/journal.pone.0256698)
Supplement: Attachment — Submitted filename: response_plosone_ipokhilenko.docx [file pone.0256698.s001.docx]

Reviewers' comments:

Reviewer's Responses to Questions

Comments to the Author

1. Is the manuscript technically sound, and do the data support the conclusions?

The manuscript must describe a technically sound piece of scientific research with data that supports the conclusions. Experiments must have been conducted rigorously, with appropriate controls, replication, and sample sizes. The conclusions must be drawn appropriately based on the data presented.

Reviewer #1: Yes

Reviewer #2: Yes

________________________________________

2. Has the statistical analysis been performed appropriately and rigorously?

Reviewer #1: Yes

Reviewer #2: I Don't Know

________________________________________

3. Have the authors made all data underlying the findings in their manuscript fully available?

The PLOS Data policy requires authors to make all data underlying the findings described in their manuscript fully available without restriction, with rare exception (please refer to the Data Availability Statement in the manuscript PDF file). The data should be provided as part of the manuscript or its supporting information, or deposited to a public repository. For example, in addition to summary statistics, the data points behind means, medians and variance measures should be available. If there are restrictions on publicly sharing data—e.g. participant privacy or use of data from a third party—those must be specified.

Reviewer #1: No

Reviewer #2: Yes

________________________________________

4. Is the manuscript presented in an intelligible fashion and written in standard English?

PLOS ONE does not copyedit accepted manuscripts, so the language in submitted articles must be clear, correct, and unambiguous. Any typographical or grammatical errors should be corrected at revision, so please note any specific errors here.

Reviewer #1: Yes

Reviewer #2: Yes

________________________________________

5. Review Comments to the Author

Please use the space provided to explain your answers to the questions above. You may also include additional comments for the author, including concerns about dual publication, research ethics, or publication ethics. (Please upload your review as an attachment if it exceeds 20,000 characters)

Reviewer #1: Overall, this is a well-written manuscript that confirms earlier findings on the relationship between trust and shared-decision making. The findings, such as the relationship between trust, SDM, and gender, are important contributions towards understanding the factors/determinants of SDM. I have no further comments on the content and recommend this manuscript for publication.

With regard to the availability of data, the authors should provide an ethical/legal justification for not making it accessible.

Author response: The authors thank the reviewer for taking time to review the manuscript and for recommending it for publication.

With regard to the availability of data, we will upload the minimal anonymized data set to replicate our study findings to the DANS EASY repository (<https://doi.org/10.17026/dans-x3y-5f6k>). This dataset will be made available upon request. Additionally, the same minimal anonymized data set is directly available upon request from prof. Judith D. de Jong (j.dejong@nivel.nl), project leader of the Dutch Health Care Consumer Panel, or the secretary of this panel (conusmentenpanel@nivel.nl). The Dutch Health Care Panel had a program committee, which supervises processing the data of the Dutch Health Care Consumer Panel and decides about the use of the data. This program committee consists of representatives of the Dutch Ministry of Health, Welfare and Sport, the Health Care Inspectorate, Zorgverzekeraars Nederland (Association of Health Care Insurers in the Netherlands), the National Health Care Institute, the Federation of Patients and Consumer Organisations in the Netherlands, the Dutch Healthcare Authority and the Dutch Consumers Association. All research conducted within the Consumer Panel has to be approved by this program committee. The committee assesses whether a specific research fits within the aim of the Consumer Panel, which is to strengthen the position of the health care user.

Reviewer #2: Overall, this is a very interesting study and will be a valuable addition to the literature on SDM and MDM. I congratulate the authors.

I have one query:

Were the survey participants asked to respond about a particular physician, e.g. their General Practitioner? If no, would the survey participants likely have multiple physicians? (In some countries patients have a General Practitioner and specialist physicians treating them at the same time).

Author response: The authors thank the reviewer for taking time to review the manuscript and for recommending it for publication. This study was conducted among the general Dutch population. The questionnaire in Dutch did not refer to any particular physician. In the Dutch healthcare system, every person is only able to be assigned to one general practitioner at a time. Therefore, we assume that the respondents provided responses regarding their general practitioner. However, we agree that in case one is treated by multiple physicians, there might be some confusion. We reflected on this point in the discussion.

“Both instruments used in this study did not refer to a particular physician (e.g. respondent’s general practitioner). This might have affected the accuracy of the responses in case some of the respondents had more than one treating physician.” (lines 347-349)

And one comment:

It would be interesting to conduct a qualitative study to understand why women with high trust are more involved in MDM compared to men with high trust – this may help with strategies to reverse men’s less than optimal involvement in MDM that you mention in the last paragraph of page 16 of the PDF.

Author response: We thank reviewer for this comment. We agree that such study could be very interesting. We added this point in the discussion.

“Employing a qualitative study design could also be interesting for investigating the reasons for why women with high trust in their physician are more involved in MDM compared to men with high trust. This could help design strategies for facilitating the involvement of men with high trust in MDM.” (lines 360-363)

________________________________________

6. PLOS authors have the option to publish the peer review history of their article (what does this mean?). If published, this will include your full peer review and any attached files.

If you choose “no”, your identity will remain anonymous but your review may still be made public.

Do you want your identity to be public for this peer review? For information about this choice, including consent withdrawal, please see our Privacy Policy.

Reviewer #1: No

Reviewer #2: No
